# Designing intervention scheme for vaccine market: a bilevel programming approach

**DOI:** 10.1007/s10696-019-09348-5

**Published:** 2019-04-06

**Authors:** Ece Zeliha Demirci, Nesim Kohen Erkip

**Affiliations:** 1 Department of Industrial Engineering and Innovation Sciences, Eindhoven University of Technology (TU/e), Eindhoven, The Netherlands; 2 Department of Industrial Engineering, Bilkent University, Ankara, Turkey

**Keywords:** OR in societal problem analysis, Supply chain management, Public-interest good, Intervention mechanism

## Abstract

Public-interest goods benefit consumers and also generate external benefits boosting societal welfare. Despite this characteristic of these goods, their level of consumption or production are generally well below the socially desirable levels without intervention. Motivated by influenza vaccine market, this paper examines the intervention design problem for a public-interest good facing yield uncertainty in production as well as inefficiencies in distribution and allocation. The proposed mechanism considers two intervention tools with the aim of resolving the inefficiencies in the system and allowing the actors to take socially desirable decisions. The first tool is to intervene so that demand level for the good is increased; we call it demand increasing strategy. The second tool aims to support the production, allocation, and distribution by investing in research and development and better planning and enhances the availability; we call this as availability increasing strategy. The intervention design problem is based on stylized demand and availability models that take into account investments made to improve them. The model suggested is experimented by a numerical study to analyze the impact of applying proposed joint mechanism in US influenza vaccine market. The results show that proposed strategy is very effectual in terms of vaccination percentages achieved and budget savings realized beyond the current practices, and the improvement in vaccination percentages is even greater when uncertainty in the system is higher. Besides, the results suggest that as long as the parameter calibration and decision problems are solved consistently, availability can be approximated by its average value when necessary.

## Introduction

Goods with positive externalities, which are referred as public-interest goods in this study, benefit consumers as well as non-paying third parties. For instance, vaccines obviously make the individuals less susceptible to a contagious disease and also they reduce the chances that non-vaccinated people will get the disease. Clearly, vaccination is good for the whole society since with only few unvaccinated individuals the transmission of the disease cannot be maintained and the risk of pandemic will be low. In a free market, this type of goods are either under-consumed or under-produced due to free-riders as well as incorrect pricing policies or ignorance of external benefits. Thus, there is a need for regulating the environment of these goods by a central authority (government or social planner) so that their consumption is raised towards a socially desirable level. Here, the main goal of the central authority is to design and fund an intervention scheme that triggers the actors of the supply chain to take decisions for the benefit of the society. In this study, we explore the problem of designing an intervention strategy for a public-interest good that has uncertainty in availability.

The motivation of this study is based on influenza vaccine supply chain. Influenza is a very well-known acute respiratory illness that circulates quickly resulting in seasonal outbreaks. World Health Organization (WHO) reports that each year the outbreaks end up with 250,000–500,000 deaths globally; additionally annual costs of outbreaks in terms of health care, lost days of work and schooling, and social disruption vary between $1 million and $6 million per 100,000 inhabitants in industrialized countries like France, Germany, and the United States (World Health Organization 2005). Annual vaccination is known to be the most effective and efficient strategy for fighting influenza to prevent morbidity and mortality. Therefore, majority of the countries carry out influenza vaccination programmes targeting nationwide coverage levels. In spite of the programmes having been implemented, current statistics show that the vaccine coverage lag behind the targeted goals in most of the developed countries (Mereckiene 2015; Centers for Disease Control and Prevention 2015a). Also, WHO reports that all countries in the world are facing influenza vaccine shortage. These problems originate from issues that are inherent to influenza vaccine supply chains.

One of the challenges of this system arises mainly due to characteristics of production process such as long production times, reformulation of vaccine composition each year, and yield issues. Majority of the production depends on flu virus grown in chicken eggs. The process starts with the announcement of the WHO Global Influenza Program on the virus strains that will be included in the forthcoming season’s vaccines. The manufacturing procedure takes approximately six months and can be summarized as follows: growing the virus in chicken eggs, harvesting the virus containing fluid from the eggs after several days, inactivating viruses, purifying, testing, and packaging (Centers for Disease Control and Prevention 2015b; Gerdil 2003). Vaccine composition is controlled each year and updated if needed due to continuous antigenic changes in the virus strains. The uncertain characteristics of the biological processes and safety and quality tests cause yield uncertainty. Specifically, either the production may result in fewer quantities or it may need extra time to end up with the desired quantities (National Vaccine Advisory Committee 2003). Besides, there are always problems of timely distribution of the usable vaccine to different areas, all causing an uncertainty in the availability of vaccine in the desired quantities at each demand point. We are going to name this phenomena as “uncertainty in availability”, caused by production yield problems that includes late production instances, as well as delays in timely distribution of vaccine that prevents effective usage (Duijzer et al. 2018; Yarmand et al. 2014). Hence, instead of produced quantity, “usable quantity” is available for effective vaccination.

Another problem is insufficient demand for reaching an effectual or socially desirable coverage level. Each vaccination decreases the infection risk for the close contacts of vaccinee and brings positive externality. This method of protection is called herd immunity. However, self-interested individuals might disregard this value while making vaccination decision with the idea of free-riding benefits from other’s vaccination. Thus, the vaccine market suffers from ignorance of externality effects and lack of incentives to get vaccinated.

The focus of this paper is designing an intervention strategy for influenza vaccines, which will decrease the effects of inefficiencies described above and encourage the channel to take a solution closer to socially optimal decision. In the strategy, we consider possibility of different efforts that will eventually increase the usable quantity. We enable this by considering a total budget, which can be used for different efforts, and solve a budget allocation problem. Formulating the problem as a budget allocation problem and solving it under a limited budget are motivated from the fact that budget is generally made up off funding from various sources (i.e. countries, organizations, and etc.) and thus the total is not fully controllable. Also, in their introduction to a special issue on health care, Brailsford et al. (2012) explicitly point out the importance of strategic decisions and budget usage in public-oriented health sector activities.

Influenza vaccines can be seen as one-time newsvendor products, as they need to be reformulated each year due to antigenic drift (Chick et al. 2008; Duijzer et al. 2018). Thus, we study the problem for a system consisting of a newsvendor firm that faces yield issues and a central authority having a fixed budget throughout the paper. For the influenza vaccine case, central authority refers to a social planner like WHO, Centers for Disease Control and Prevention (CDC), etc. or a government. The role of the central authority is to coordinate the expenditures limited by the budget and maximize the social welfare. A critical issue while designing an intervention mechanism is to choose the intervention tools to be considered and their associated effects.

One alternative is making investment in demand increasing strategies. We assume that this investment is devoted to any attempt that will promote vaccination. In practice, these may correspond to subsidizing vaccine cost, public education, media campaigns to inform those at high risk, expanding access to vaccination services (i.e. via pharmacy, immunization centers near schools and worksites, home visits), organizing school vaccination programs, and establishing client reminder and recall systems (Centers for Disease Control and Prevention 2015a). A different example to demand increasing strategies can be given based on a recent news discussing low immunization rates for HPV (The New York Times 2015). Current policy issues and lack of MD endorsement (and recommendation) are found to be responsible for the shortfall in the usage of these vaccines. For this case mentioned in the news, organizing information briefing for doctors can be a good example for demand increasing strategies so that they will make timely recommendations to children for receiving the vaccine.

Another intervention tool is availability increasing strategy through which usable quantity of the production decision can be increased. The uncertainty associated with the availability of flu vaccines has two causes which are output quantity due to random yield and delivery timing (Dai et al. 2016). The former dimension of uncertainty is mainly due to current production technologies that have several limitations such as yield issues, production capacity, and ability to accelerate production in case of pandemic. These inefficiencies can be overcome through financial support to manufacturer to increase yield and thus availability. Deo and Corbett (2009) also highlight that it is required to subsidize research on production processes instead of only implementing immunization programs. This can be done by giving funding to manufacturer to share his risk or investing in new production technologies. For instance, U.S. government and the Crucell Corporation have been investing in cell based technology which promises to provide more flexibility and has the potential to reduce lead times and yield uncertainty (Williams 2016). The U.S. National Institutes of Health (NIH) granted $9.5 million to ID Biomedical to develop this technology. In 2016, FDA has approved cell isolated vaccine viruses and the only cell based flu vaccine has been licensed (Centers for Disease Control and Prevention 2019). The latter dimension of uncertainty in the availability includes timing inefficiencies in production and delivery timing (Duijzer et al. 2018). Several examples from US can be found. An early example can be given as 2000–2001 season during which influenza vaccine coverage has been realized 16% lower than the previous season while leaving 7.5 million doses unused (Dai et al. 2016). The reason behind has been explained by the unavailability of vaccines during peak demand times of October and November which results with the cancellation of vaccination campaigns. A more recent example is delayed shipments during 2014–2015 season leading to shortage during peak demand time. These examples bring out the idea of removing these issues by better planning and support to manufacturer to encourage him to initiate at risk early production prior to the design freeze.

In brief, in this study we analyze an intervention mechanism composed of the two intervention tools described above. We note that the proposed joint mechanism’s objectives are parallel with the objectives of GAP (Global action plan for influenza vaccines), which is carried on by WHO (World Health Organization 2015). GAP is an exhaustive project aimed at achieving higher vaccine usage, improving production capacity, and research and development.

To analyze the above mentioned framework, we build a bilevel programming model, which enables us to integrate manufacturer’s point of view into the central authority’s decision making process. We assume that demand and availability are correlated random variables without loss of generality and follow bivariate lognormal distribution. Bivariate lognormal distribution is quite general as one can represent distributions with different shapes and values of coefficient of variation. We express demand and availability as functions of corresponding investments made to improve them. Under the lognormal demand and availability models proposed, given central authority’s decisions we obtain closed form expression for the optimal quantity to be produced by the manufacturer. We also characterize the optimal solution for specific forms of authority’s objective function, and demand and availability functions.

The framework considered in the study is abstract when compared with the difficulties encountered in a real environment. As a result, we include some numerical analyses to reflect the usability of such a model. To realize any analysis we set up a case study with parameters reflecting an actual instance. Additionally, we plan for some specific analyses (other than standard sensitivity) to understand the reflection of the numerical results on the decision making processes. Finally, bringing all together, we aim to accumulate information which will be instrumental to persuade the decision makers to utilize such approaches. With these in mind we complete our presentation with numerical experiments.

The numerical experiments rely on available estimates from studies on influenza vaccine supply chains in literature and CDC’s statistics on flu vaccination coverage in US. We propose a parameter calibration procedure based on available information. Our test results indicate that considerable improvement can be achieved by the joint policy. Moreover, we compare the results with the case where imperfect availability is approximated by a deterministic function dependent on investment made to improve it. The solutions show that the vaccination coverage obtained with deterministic availability function are very close to the ones achieved under availability uncertainty if the demand function’s parameters are calibrated accurately for each version. Finally, we add a discussion and our findings on the selection of total budget level to support decision makers. Note that the production processes in many industries face availability uncertainty such as agriculture, biofuel production, mining, and etc. Although the framework of the study is inspired from influenza vaccines, it can be applied to other goods with similar characteristics.

In the conclusion section, we present a summary of our results. We also discuss the usability of this model for practitioners using the results of the technical parts, as well as the numerical analyses.

## Literature review

Recent studies in operations management have examined the design of incentives in public-interest goods context. The incentives considered are generally in the form of rebates (i.e. refund of money awarded to consumers of that particular product) or subsidies (i.e. payment to the firm that manufactures and/or sells the product for every unit produced/ordered). There are a number of studies that consider a single intervention tool in order to motivate the adoption of this type of goods, which are Lobel and Perakis (2011), Cohen et al. (2015), and Chemama et al. (2014). Lobel and Perakis (2011) consider the use of rebates in order to reach a specified adoption target for solar photovoltaic technology. Cohen et al. (2015) investigate the impact of demand uncertainty on the optimal rebate amount as well as governmental expenditure, supplier’s profit, and consumer surplus. Chemama et al. (2014) develop a two-period model to study the effect of committing to a rebate amount along two periods versus adjusting it among the periods on the risk sharing between government and supplier. On the other hand, there are studies on intervention design and its impacts on the societal and players’ outcomes in a setting including multiple intervention tools. Raz and Ovchinnikov (2015) and Taylor and Xiao (2014) employ rebates and subsidies for coordinating the market. Raz and Ovchinnikov (2015) analyze the problem via three different mechanisms (i.e. joint mechanism consisting both tools and two simplified mechanisms including one of the tools) and show that joint mechanism can coordinate the system, whereas simplified mechanisms can coordinate either price or quantity. In contrast, Taylor and Xiao (2014) explores the problem for a long shelf life product like malaria drugs and show that the donor should only subsidize the firm at the optimal scheme. In Demirci and Erkip (2017), central authority uses a joint mechanism composed of investment in demand increasing strategies and rebates for regulating the system. They present a model that decides on the optimal budget amount and details of intervention mechanism. One possible example of public-interest good is home care service with a lot of inference to possible strategic and tactical level plans as described by Matta et al. (2014). They present a model for home care organisations from an operations management perspective. Additionally, as a solution methodology we use a similar approach employed by Demirci and Erkip (2017). However, the characteristic of this problem is quite different as this one includes an explicit budget consideration, as well as random yield in addition to random demand and different intervention tools.

At the same time, there is an increasing interest in vaccine supply chain related decision problems in OR/OM literature. Duijzer et al. (2018) presents a structured overview of vaccine supply chain literature by classifying the studies depending on the supply chain component under consideration, which are composition, allocation, distribution, and production. The studies on vaccine composition investigate the problem of which virus strains to include in the vaccine for either influenza or HIV vaccines. Examples for the ones focusing on influenza vaccines are Kornish and Keeney (2008), Cho (2010), and Özaltın et al. (2011), and examples for HIV are Porco and Blower (1998) and Maher and Murray (2016). Besides, there are also studies focusing on epidemic dynamics of influenza, which can be utilized for making preparatory planning. One recent example is Lee and Shin (2016). The vaccine allocation problem decides on for which groups vaccination will be administered. As the problem is a higher level decision problem and quite general, it has been studied for various types of diseases and cases. Some recent studies are Samii et al. (2012), Tanner and Ntaimo (2010) and Yarmand et al. (2014). Vaccine distribution problem includes several operational problems. One is determining how to distribute the vaccines, i.e. via fixed locations (or point of dispensing) or mobile facilities. Each option brings out new questions like facility location and layout and staffing decisions if point of dispensing is chosen and routing problems otherwise. The other operational problem is to decide on size and location of vaccine inventories. Examples of studies dealing with these problems are Jacobson et al. (2006), Halper and Raghavan (2011), and Ramirez-Nafarrate et al. (2015). The studies which are closely related to our work fall into the production category. This cluster of papers has been devoted to find schemes to coordinate the vaccine market. We will only review the ones which focus on influenza vaccines in the remaining part of this section.

The inefficiencies of a flu vaccine supply chain emanating from operational issues on the supply side and negative externality effect on the demand side mostly have not been addressed concurrently in prior studies. A group of studies focus on only supply uncertainty and its impact on social welfare. Chick et al. (2008) present first integrated supply chain/health economics model for a system consisting of a monopolistic manufacturer that sells vaccines to a government. Based on their results they investigate that manufacturer bears all of the production risks due to lack of coordination, which results in shortfall of vaccines. Hence, they derive a variant of cost sharing contract, which provides an incentive to the manufacturer to produce social optimum quantity. In Chick et al. (2016), the major concern is to design a contract that will align incentives of government and manufacturer as in Chick et al. (2008), but they consider an environment with asymmetric information about production uncertainty and include the possibility to fulfill the shortfall demand at a higher cost after the delivery date. Mamani et al. (2013) extend the study of Chick et al. (2008) to a scenario with multiple governments and risk of disease transmission across borders. They design a contractual agreement among governments that will enhance global health outcomes. Using a Cournot competition model, Deo and Corbett (2009) argue that the limited number of entrants in a vaccine market, vaccine undersupply and low society surplus can be explained by yield uncertainty inherent to this environment. In all of these studies demand is exogenous to their models; however the consumer behavior (reflecting negative network externality effect) is also incorporated in this context more recently. Briefly, they assume that consumers’ decision of whether to uptake vaccine depends on the vaccinated fraction of the population. Mamani et al. (2012) show that in an oligopolistic vaccine market, a fixed subsidy should be administered to consumers so that a socially optimal immunization rate can be reached. However, they ignore the impact of yield uncertainty on the subsidy design and social welfare. Later, Adida et al. (2013) study a similar problem in a setting that includes consumer behavior as well as yield uncertainty, and show that a fixed two-part subsidy scheme is not sufficient to coordinate even the monopoly market. They propose a two-part menu of subsidies that includes a subsidy dependent on coverage level given to the consumers and a unit production payment to the manufacturer in order to eliminate the market inefficiencies. In contrast to previous studies, Arifoğlu et al. (2012) do not formalize the setting as an incentive design problem, but instead explore the implications of demand side versus supply side interventions on the manufacturer’s decision and societal outcome under different conditions. In addition to yield uncertainty considered in the articles described below, Yarmand et al. (2014) shows that allocation problem and timing of distribution significantly affects the issue of availability as mentioned in the introduction.

Unlike the existing studies in the literature, our model is a budget allocation problem and further decides on the quantity to be produced and intervention scheme under uncertainty in both demand and availability. Also, our work is differentiated from previous work in terms of using strategic intervention tools simultaneously and their effects on the system dynamics, modelled in a very general way. However, we do not take into account epidemiological details and just consider the initial preventive stage of vaccination. In other words, we simply treat the problem as a single stage problem, following similar others in the literature. This makes our budget allocation framework applicable to other types of goods with resembling characteristics.

## Model and assumptions

This section presents the decision model to determine the allocation of budget among intervention tools, and demand and availability models.

### Decision model

We model intervention design problem for the context discussed by bilevel programming. Bilevel programming involves problems of two independent hierarchical decision makers within a single instance, problem of one becoming part of constraints of the other one. This type of problems includes an upper-level decision maker or leader and a lower-level decision maker or follower. Each player attempts to optimize its own objective, but they are affected from each other’s decision. A distinguishing property of this programming is that leader can influence follower’s decision, but cannot dominate completely [see Colson et al. (2007) and Bard (1998) for more comprehensive information].

In our case, the central authority is the leader, who is interested in maximizing the social welfare, whereas newsvendor firm is the follower with the objective of maximizing expected profit. Newsvendor firm faces imperfect yield and uncertainty in availability due to characteristics of production and distribution processes. We model uncertainty in availability with stochastically proportional approach, which is widely studied in the literature [see Yano and Lee (1995) for a detailed review of approaches solving lot sizing problem with random yield]. This approach is applicable to systems like influenza vaccine production system, in which availability uncertainty emanates from inflexibility of production system to adapt to changes in the environment or material, distribution and allocation.

Central authority intervenes in the system through a joint mechanism composed of two intervention tools: (1) investment made in demand increasing strategies, $B_d$, and (2) investment made in availability increasing strategies, $B_y$. Any effort aimed at increasing demand will shift the demand distribution, and similarly investment made in availability increasing strategies will affect availability distribution. Thus, mean demand and mean availability increase as investment made in demand increasing and availability increasing strategies increase, respectively. We assume that the social welfare/utility is assessed by a function of manufacturing quantity, which in turn is a function of the intervention mechanism (i.e. $B_d$ and $B_y$). The reason behind this is that the main goal is to increase the adoption level in this environment. The adoption level is closely related with the quantity produced as the self-interested individuals decide whether to search for vaccine by knowing the available quantity (Arifoğlu et al. 2012; Arifoğlu 2012). Note that adoption level also considers the inefficiency in the allocation, as well as timely distribution of the produced amount.

**Table 1:** Notation

*r*	Unit selling price
*c*	Unit manufacturing cost
*s*	Unit salvage value
$B_d$	Investment made in demand increasing strategies
$B_y$	Investment made in availability increasing strategies
*B*	Total available budget of central authority
$Q(B_d, B_y)$	Manufacturing quantity of the manufacturer
*u*(.)	Utility function of the central authority
$P(Q,B_d,B_y)$	Profit function of the manufacturer
$D(B_d)$	Random variable denoting demand
$Y(B_y)$	Random variable denoting availability

Let *r*, *c*, *s* be unit selling price, manufacturing cost, and salvage value respectively. *u*(.) denotes the utility of the central authority (reflecting public view), which is a function of the production quantity *Q*. Note that production quantity, in turn is a function of the budgets made available for different strategies. We assume a strictly concave utility as a function of *Q* for every $B_d$ and $B_y$. A summary of the notation is presented in Table 1 and the bilevel programming formulation of the problem (*BLP*) is given below:

1 $$\begin{aligned} \max \limits _{B_d,B_y}&\quad u(Q(B_d, B_y)) \end{aligned}$$

2 $$\begin{aligned}\text{ s.t. }&\quad B_d+B_y\le B
\end{aligned}$$

3 $$\begin{aligned} \quad \text{  } &B_d, B_y \ge 0\end{aligned}$$

4 $$\begin{aligned} & Q(B_d, B_y)= arg\,max_Q\,P(Q,B_d,B_y). \end{aligned}$$

where $P(Q,B_d,B_y)$ is the profit function of the newsvendor firm and is given by:

$$\begin{aligned} P(Q,B_d,B_y)=\, & {} rY(B_y)Q(B_d,B_y)1_{\left\{ Y(B_y)Q(B_d,B_y)\le D(B_d)\right\} }\\&+rD(B_d)1_{\left\{ Y(B_y)Q(B_d,B_y)\ge D(B_d)\right\} }+s(Y(B_y)Q(B_d,B_y)\\&-D(B_d))1_{\left\{ Y(B_y)Q(B_d,B_y) \ge D(B_d)\right\} }-cQ(B_d,B_y) \end{aligned}$$

with $1_A=1$ if *A* is true, and 0 otherwise. $D(B_d)$ and $Y(B_y)$ are the random variables denoting demand and availability, respectively. Note that they are dependent on the investment amount made in to improve them.

The usable quantity that will be received at the end of processes is *YQ* as we explore the model for stochastically proportional availability. Also, note that we expect $B_d$ and $B_y$ affecting the distributions of demand and availability individually and through their possible correlation. Specifically, the lower level problem is a newsvendor model with dependent random availability and demand. A recent study by Okyay et al. (2014) also studied a newsvendor problem with similar characteristics from the perspective of an inventory manager who decides on how much to order. However, we consider the problem for a manufacturer and thus the cost is not only incurred for the usable quantity *YQ* but for *Q*. Thus, the optimality condition of our problem would be different than the presented one in Okyay et al. (2014).

In this problem, the central authority goes first and determines the investment amounts made in intervention tools, and later in view of his decisions manufacturer decides on production quantity. Equations (1)–(3) express the central authority’s problem, while (4) corresponds to the manufacturer’s problem. The objective of the central authority is to find the utility maximizing investment amounts, $B_d$ and $B_y$. The selection of $B_d$ and $B_y$ affects the solution and objective function of the manufacturer by affecting the distributions used to evaluate $P(Q,B_d,B_y)$, and in turn manufacturer’s decision influences the central authority’s utility. Constraint (2) imposes budget constraint on the total money invested for demand and availability increasing strategies. Equation (3) guarantees non-negativity of $B_d$ and $B_y$. Lastly, Eq. (4) reflects the newsvendor problem under random availability for given $B_d$ and $B_y$.

### Analysis of the decision model

Primary approach for solving bilevel optimization problems is replacing lower level problem by its first order conditions. Thus, we start our analysis by the lower level problem.

We find out that the expected profit maximizing $Q(B_d,B_y)$ occurs at

5 $$\begin{aligned} E\left[ Y(B_y)1_{\left\{ D(B_d)\le Y(B_y)Q(B_d,B_y)\right\} }\right] =\frac{rE[Y(B_y)]-c}{r-s}. \end{aligned}$$

The details of derivation can be found in “Appendix 1”.

#### *Remark 1*

The following conditions should hold so that it is economic to manufacture for the newsvendor:

6 $$\begin{aligned} rE[Y(B_y)]> & {} c, \end{aligned}$$

7 $$\begin{aligned} sE[Y(B_y)]< & {} c. \end{aligned}$$

Condition (6) guarantees that the average revenue is higher than the unit production cost, whereas condition (7) ensures that the unit production cost is greater than the average salvage value.

#### **Lemma 1**

$E[P(Q,B_d,B_y)]$*is strictly concave inQfor every*$B_d$*and*$B_y$. *This assures that the solution to the lower level problem is unique, implying that the lower level problem can be replaced by its first order condition (by p. 308 of* Bard 1998).

Accordingly, BLP can be written as the following single-level mathematical program (*SP*):

8 $$\begin{aligned}&\max \limits _{B_d,B_y}&\qquad u(Q(B_d, B_y)) \end{aligned}$$

9 $$\begin{aligned}&\text{ s.t. }&\qquad B_d+B_y\le B \end{aligned}$$

10 $$\begin{aligned} \qquad \qquad \quad &B_d, B_y \ge 0\end{aligned}$$

11 $$\begin{aligned} \qquad &(5). \end{aligned}$$

Now, we obtain a relatively easy to solve standard nonlinear program. In this formulation, the central authority optimizes utility/social welfare subject to her constraints and optimal decision of manufacturer.

### Representation of demand and availability

We use lognormal distribution to represent demand and availability as various distributional shapes can be represented by it. The distribution is specified with two parameters, one of which being a shape parameter and the other being a scale parameter (Law et al. (1991)). These two parameters give the flexibility to determine whether the distribution is right or left skewed. Also, as an example having a pdf with a long right tail enables us to cover almost deterministic availability case in case it models the availability or makes high demand values to have almost zero probability if it is used to model demand.

We assume that demand comes from a family of lognormal distributions with parameter $B_d$. Once $B_d$ is set, the demand distribution will be known accordingly. For generality, we assume that $B_d$ will affect both the mean and standard deviation of the demand. Let $H_D(B_d)$ and $V_D(B_d)$ be functions of $B_d$. Then, we can represent the demand by

$$\begin{aligned} D(B_d)=H_D(B_d)e^{-\frac{1}{2}\sigma _{D}^2 V_D(B_d)^{2}+\sigma _D V_D(B_d) W_D}, \end{aligned}$$

where $W_D$ has standard normal distribution and $\sigma _D V_D(B_d)$ is a parameter of the distribution. It can be verified that $E[D(B_d)]=H_D(B_d)$, where $H_D(B_d)$ is a function of $B_d$, and $Var(D(B_d))=H_D(B_d)^2(e^{\sigma _{D}^2 V_D(B_d)^{2}}-1)$.

#### *Remark 2*

If $H_D(B_d)$ is an increasing function of $B_d$ and $V_D(B_d)$ is a non-increasing function of $B_d$, there will be a first order stochastic dominance order between cdfs of demand. Specifically, the cdf with higher $B_d$ value will stochastically dominate all others with smaller $B_d$.

One can write coefficient of variation of demand as $cv_D(B_d)=\sqrt{e^{\sigma _{D}^2 V_D(B_d)^2}-1}$. Hence, an increase in $B_d$ will not increase the $cv_D$ value. Note that the assumptions on the form of the functions are very general and intuitively require that any additional budget devoted to increase demand will increase the mean, but not increase (likely to decrease) the coefficient of variation of demand.

We construct the availability function with a similar reasoning as in demand function. We use a lognormal availability function dependent on parameter $B_y$.

$$\begin{aligned} Y(B_y)=H_Y(B_y)e^{-\frac{1}{2}\sigma _{Y}^2 V_Y(B_y)^2+\sigma _Y V_Y(B_y) W_Y}, \end{aligned}$$

where $W_Y$ has standard normal distribution and $\sigma _Y V_Y(B_y)$ is a parameter of the distribution. As in the demand function, $E[Y(B_y)]=H_Y(B_y)$, where $H_Y(B_y)$ is an increasing concave function in $B_y$ and takes values in the interval [0,1], and $Var(Y(B_y))=H_Y(B_y)^2(e^{\sigma _{Y}^2 V_Y(B_y)^2}-1)$. Similarly, coefficient of variation is given by $cv_Y(B_y)$= $\sqrt{e^{\sigma _Y^{2}V_Y(B_y)^2}-1}$. Also, first order stochastic dominance order holds for the availability model as in demand case. Note that availability is allowed to take values larger than 1, although the probability of such events is usually small depending on the selected parameters and reasonable budget levels considered.

We assume that demand and availability can be jointly lognormally distributed. Specifically, natural logarithms of random variables *D* and *Y* have bivariate normal distribution with means $ln (H_D(B_d))-\sigma _D^{2} V_D(B_d)^2/2$ and $ln(H_Y(B_y))-\sigma _Y^{2} V_Y(B_y)^2/2$, and standard deviations $\sigma _D V_D(B_d)$ and $\sigma _Y V_Y(B_y)$, respectively, and correlated by $\rho$. Specifically, $(W_D, W_Y)$ has a standard bivariate normal distribution with correlation coefficient $\rho$, $E[W_D, W_Y]=\rho$.

Recall that $B_d$ denotes the investment amount allocated to demand increasing strategies, whereas $B_y$ reflects the investment amount allocated to availability improving strategies in our model. Any effort targeted to achieve higher demand attracts more consumers for vaccine uptake and thus shifts the mean demand upwards. Besides, investment devoted to availability increasing strategies decreases the risks included in the manufacturing process and improves the yield, as well as decreases the inefficiencies in vaccine distribution. Thus, an increase in the investment amounts $B_d$ or $B_y$ leads to a first order stochastic dominance order between cdfs of demand or availability, respectively as described in Remark 2. Note that we can also envision a correlation between the uncertain components of demand and availability variables. Positive correlation indicates that higher demand motivates the manufacturer to improve availability, as well as improved availability may result in a tendency for more demand. On the other hand, it is harder to justify negative correlation though. One possible explanation might be to consider a situation where knowledge of higher levels of availability dampening the artificial demand amplification caused by the previous unacceptable experiences.

## Analysis of the model

In the later sections of the study, we assume specific forms for $H_D(B_d)$ and $H_Y(B_y)$. Although there are not many studies on investment response functions, an advertising response function is generally assumed to be concave in advertising expenditure in previous studies (e.g. Khouja and Robbins 2003; Lee and Hsu 2011; Arcelus et al. 2006). We follow the same assumption in our analysis. Under a concave response function, as the money invested increases the mean demand and mean availability also increase but with a monotonically diminishing rate. In our analysis, we use the mean demand function analyzed in Khouja and Robbins (2003), which is given below:

$$\begin{aligned} H_D(B_d)=\mu +\mu w B_d^{\alpha } \end{aligned}$$

where $\mu$ is the initial mean demand (i.e. mean demand before intervention), and *w* and $\alpha$ ($w \ge 0$, $0 \le \alpha \le 1$) are constants that reflect effectiveness of demand increasing strategy. For a fixed $w > 0$, mean demand increases at a faster rate for larger values of $\alpha$. When $w=0$, mean demand is not influenced by demand increasing strategies.

One possible form for $H_Y(B_y)$ is as follows:

$$\begin{aligned} H_Y(B_y)=\frac{kB_y+1}{kB_y+a},{\text { for }}a>1{\text { and }}k\ge 0. \end{aligned}$$

As $B_y\rightarrow 0, H_Y(B_y) \rightarrow \frac{1}{a}$ and as $B_y\rightarrow \infty , H_Y(B_y) \rightarrow 1$. 1 / *a* gives the initial availability (availability rate without intervention) and *k* is a measure for the efficiency of the availability improving technology.

For further analysis we consider $V_D(B_d)=1$ and $V_Y(B_y)=1$. Both cases will yield family of demand distributions that have equal coefficient of variations. We think that this represents a pessimistic view for the use of budgets, as additional budget is not effective in changing the variability represented by the coefficient of variation values.

### Manufacturer’s problem

We use the properties of lognormal random variables to derive $E[Y1_{\left\{ D\le YQ\right\} }]$ (see “Appendix 1” for the details) and express it as follows:

12 $$\begin{aligned}&E\left[ Y(B_y)1_{\left\{ D(B_d)\le Y(B_y)Q(B_d,B_y)\right\} }\right] \nonumber \\&\quad =H_Y(B_y) \Phi \left( \frac{ln(Q(B_d,B_y))+ln(H_Y(B_y))-ln(H_D(B_d))+\sigma _{Y}^{2}/2+\sigma _{D}^{2}/2-\rho \sigma _Y \sigma _D}{\sqrt{\sigma _{Y}^{2}+\sigma _{D}^{2}-2\rho \sigma _Y \sigma _D}}\right) \end{aligned}$$

Solving (5) and (12) for *Q* gives:

13 $$\begin{aligned} Q(B_d,B_y)=e^{\left[ \Phi ^{-1}\left( \frac{rH_Y(B_y)-c}{(r-s)H_Y(B_y)}\right) \sqrt{\sigma _{Y}^{2}+\sigma _{D}^2-2\rho \sigma _Y\sigma _D}-\ln (H_{Y}(B_y))+\ln (H_D(B_d))-\sigma _{Y}^2/2-\sigma _D^{2}/2+\rho \sigma _Y\sigma _D\right] }, \end{aligned}$$

where $\Phi ^{-1}(.)$ denotes the inverse of the standard normal cdf.

Along with the fact that the argument of $\Phi ^{-1}(.)$ should be between 0 and 1, we deduce the following conditions on $H_Y(B_y)$ from (13):

14 $$\begin{aligned} H_Y(B_y)> & {} \frac{c}{r}, \end{aligned}$$

15 $$\begin{aligned} H_Y(B_y)< & {} \frac{c}{s}. \end{aligned}$$

Note that these conditions are similar to the ones given in Remark 1. It is critical to check whether the condition given by equation (14) is always satisfied or not while solving the model. For the specific form $H_Y(B_y)=\frac{kB_y+1}{kB_y+a}$, this condition is always satisfied if $r > ac$. Actually, this condition on the model parameters is a natural assumption for the newsvendor so that it is economic to operate. In the opposite case (if $r<ac$), the intervention mechanism is able to make the system economic by investing on availability increasing strategies, and consequently increasing the availability. Note that in this case, there is a minimum budget requirement constraint for $B_y$, i.e. $B_y > \frac{ac-r}{k(r-c)}$, that will enable the manufacturer to operate.

#### *Remark 3*

If $$\sqrt{\sigma _{Y}^{2}+\sigma _{D}^2-2\rho \sigma _Y\sigma _D} > 0.3989 \left( \frac{r-s}{c} \right)$$ , then $$\frac{\partial Q(B_d,B_y)}{\partial B_y} > 0$$.

See “Appendix 1” for the proof.

**Fig. 1:**
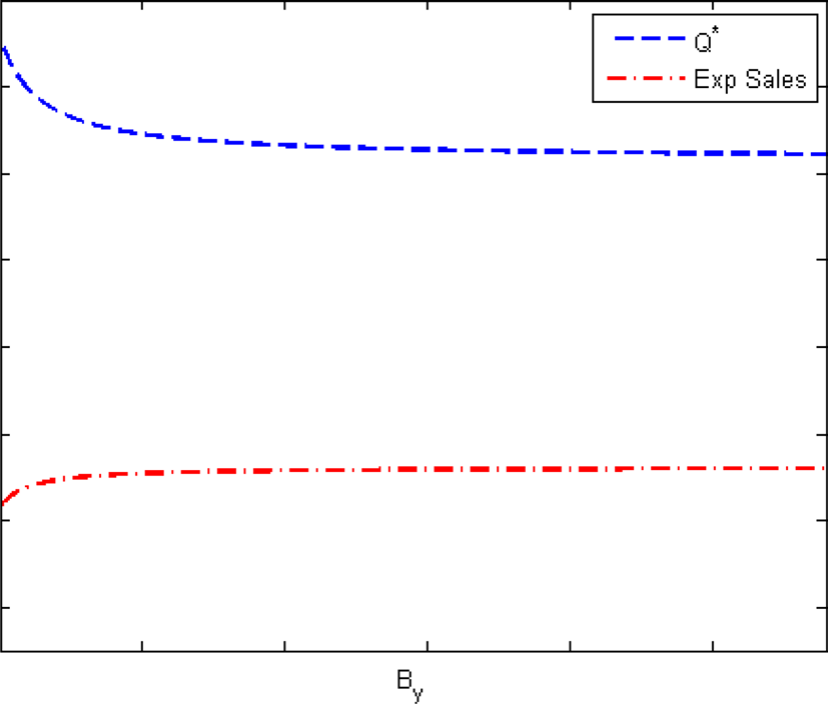
Example for Remark 3 (with parameter values of $r=15$, $c=3$, $s=0$, $cv_D=cv_Y=0.5$, $\rho =-0.5$, $a=1.43$, $w=0.05$, $k=10^{-6}$, $B_d=10^6$)

Remark 3 states a sufficient condition to ensure an increase in $B_y$ will in turn increase *Q*. However, at the same time, it implies that when uncertainty is low or when the system is very profitable, availability increasing strategies may have no effect or negative effect on *Q*. Note that this does not necessarily mean that expected sales is decreasing. Figure 1 depicts an example for this case. Briefly, the figure illustrates optimal manufacturing quantity and expected sales as a function of $B_y$ for a given value of $B_d$. The intuition is that the improvement in availability does not necessarily imply an increase in targeted quantity, $Q^*$, since the realized quantity will be more by manufacturing with a higher availability factor.

### Further results when utility function is expected sales or any increasing function of expected sales

Motivated by the vaccine market’s environment, we use the expected sales value to assess the utility for this problem. Note that one can use any objective function that will reflect societal benefit. The governments’ or social planner’s main goal is to reach or get closer to socially optimal level of vaccine coverage, which is usually stated as a coverage rate. Also, the objective has been used as coverage rate in previous studies like (Adida et al. 2013). Therefore, for budgets that are not extremely large the expected sales will be a realistic objective function consistent with the current market environment. We discuss the effect of the budget size in Sect. 5.2.4. In the remaining part of the study, we continue our analysis by taking the objective as expected sales.

Expected sales with respect to our demand and availability models can be expressed as follows (see “Appendix 1” for details of derivation) :

16 $$\begin{aligned}&E\left[ min \left\{ Y(B_y)Q(B_d,B_y), D(B_d) \right\} \right] \nonumber \\&\quad = H_D(B_d)\Phi \left( \frac{ln(Q(B_d,B_y))-ln(H_D(B_d))-\sigma _D^{2}/2+ln(H_Y(B_y))-\sigma _Y^{2}/2+\rho \sigma _D \sigma _Y}{\sqrt{\sigma _D^2+\sigma _Y^2-2\rho \sigma _D \sigma _Y}}\right)&\nonumber \\&\quad +H_Y(B_y)Q(B_d,B_y) \nonumber \\& \qquad \Phi \left( \frac{-ln(Q(B_d,B_y))+ln(H_D(B_d))-\sigma _D^{2}/2-ln(H_Y(B_y))-\sigma _Y^{2}/2+\rho \sigma _D \sigma _Y}{\sqrt{\sigma _D^2+\sigma _Y^2-2\rho \sigma _D \sigma _Y}}\right) \end{aligned}$$

Furthermore, we state the below proposition using the fact that expected sales is an increasing (and strictly concave) function of *Q*.

#### **Proposition 1**

*Consider all the conditions stated by Remark* 1, *Remarks* 2*and* 3*are satisfied, so thatQis an increasing function of both*$B_d$*and*$B_y$. *Under these conditions, all of the budget will be used at the central authority’s optimal intervention scheme, i.e.*$B_{d}^{*}+ B_{y}^{*}=B$*holds at the optimal solution*$(Q^*, B_{d}^{*}, B_{y}^{*} )$.

*See* “Appendix 1” *for the proof*.

Now, using expected sales as the objective function and utilizing the optimal solution characteristics of the problem for a strictly concave and monotone increasing utility function of *Q* [i.e. Proposition 1 and Equation (13)], we express the problem in terms of a single variable. The formulation is as follows:

17 $$\begin{aligned} \max \limits _{B_y}&\quad H_D(B-B_y)\Phi \left( \Phi ^{-1} \left( \frac{rH_Y(B_y)-c}{(r-s)H_Y(B_y)}\right) -\sqrt{\sigma _D^2+\sigma _Y^2-2\rho \sigma _D\sigma _Y} \right) \nonumber \\&\qquad \qquad + H_Y(B_y)\left( \frac{c-sH_Y(B_y)}{(r-s)H_Y(B_y)}\right) \nonumber \\&\quad \times e^{\left[ \Phi ^{-1}\left( \frac{rH_Y(B_y)-c}{(r-s)H_Y(B_y)}\right) \sqrt{\sigma _{Y}^{2} +\sigma _{D}^2-2\rho \sigma _Y\sigma _D}-\ln (H_{Y}(B_y))+\ln (H_D(B-B_y)) -\sigma _{Y}^2/2-\sigma _D^{2}/2+\rho \sigma _Y\sigma _D\right] } \end{aligned}$$

18 $$\begin{aligned} \text{ s.t. }&\quad 0 \le B_y\le B \end{aligned}$$

We conduct a set of numerical experiments to study the structure of the problem for the following mean demand and availability functions; $H_D(B-B_y)=\mu +\mu w (B-B_y)^{\alpha }$ and $H_Y(B_y)=\frac{kB_y+1}{kB_y+a}$. In the examples solved, we observe that the objective function is concave with respect to $B_y$. However, we are not able to prove the concavity in general.

### Approximate representation of the objective function with expected availability

In this subsection, we define a new objective function that is $E[min \left\{ E[Y(B_y)]Q(B_d,B_y), D(B_d) \right\} ]$, which is alike expected sales, and compare its value with the former one for given intervention design. Specifically, we replace $E[min \left\{ Y(B_y)Q(B_d,B_y), D(B_d) \right\} ]$ term with $E[min \left\{ E[Y(B_y)]Q(B_d,B_y), D(B_d) \right\} ]$. We consider this situation as one would see such cases in practice. The new objective function for bivariate lognormal distribution can be written down as follows:

19 $$\begin{aligned}&E\left[ min \left\{ E[Y(B_y)]Q(B_d,B_y), D(B_d) \right\} \right] \nonumber \\&\quad = H_D(B_d)\Phi \left( \frac{ln(E[Y(B_y)]Q(B_d,B_y))-ln(H_D(B_d))-\sigma _D^{2}/2}{\sigma _D}\right) \nonumber \\&\qquad +H_Y(B_y)Q(B_d,B_y) \Phi \left( \frac{-ln(E[Y(B_y)]Q(B_d,B_y))+ln(H_D(B_d))-\sigma _D^{2}/2}{\sigma _D}\right) \end{aligned}$$

See “Appendix 1” for the derivation.

#### **Proposition 2**

*Given*$B_d$*and*$B_y$, *the following relations hold between the two objective function candidates:*

$$\begin{aligned} \begin{array}{llll} \text{ If } \rho \le 0,&{}\quad E\left[ min \left\{ E[Y(B_y)]Q(B_d,B_y), D(B_d) \right\} \right] \\ &{}\qquad>E\left[ min \left\{ Y(B_y)Q(B_d,B_y), D(B_d) \right\} \right] ,\\ \text{ Otherwise },&{}\quad E\left[ min \left\{ E[Y(B_y)]Q(B_d,B_y), D(B_d) \right\} \right] \\ &{}\qquad =E\left[ min \left\{ Y(B_y)Q(B_d,B_y), D(B_d) \right\} \right] \quad \text {if}\quad \sigma _Y=2\rho \sigma _D\\ &{}\quad E\left[ min \left\{ E[Y(B_y)]Q(B_d,B_y), D(B_d) \right\} \right] \\ &{}\qquad>E\left[ min \left\{ Y(B_y)Q(B_d,B_y), D(B_d) \right\} \right] \quad \text {if}\quad \sigma _Y>2\rho \sigma _D\\ &{}\quad E\left[ min \left\{ E[Y(B_y)]Q(B_d,B_y), D(B_d) \right\} \right] \\ &{}\qquad<E\left[ min \left\{ Y(B_y)Q(B_d,B_y), D(B_d) \right\} \right] \quad \text {if}\quad \sigma _Y<2\rho \sigma _D \end{array} \end{aligned}$$

*See* “Appendix 1” *for the proof*.

In general, the objective function value with $E[Y(B_y)]$ replacing the random availability variable will be either an over or under-estimate of the expected sales value. The conditions indicate that if demand and availability are uncorrelated or negatively correlated, $E[min \left\{ E[Y(B_y)]Q(B_d,B_y), D(B_d) \right\} ]$ overestimates the expected sales value.

## Numerical analysis

In this section, we present a numerical study using demand and availability functions explained in Sect. 4 and model (SP) given in Sect. 4.2 with the objective of maximizing expected sales in order to gain insights about the impact of applying joint mechanism on US influenza vaccine market. The numerical study is conducted based on available information from prior studies on influenza vaccine supply chain and US influenza vaccine market. We investigate how the proposed intervention mechanism proceeds and provide a detailed discussion of the results. Before continuing with the numerical results, we first explain how we set up our parameter sets.

### Data set and calibration procedure for demand model’s parameters

We choose the cost parameters and availability information from prior related studies in the literature, the details can be found in Table 2. The remaining parameters to calibrate are related to the demand and availability functions, namely *k*, $cv_d$, $cv_y$, $\rho$, $\mu$, *w*, and $\alpha$. Our available information allows us to calibrate only two of these variables given the others. With the limited available information, our approach is then to set some of these parameters to certain values and calibrate the remaining.

**Table 2:** Base parameter set

Parameter	Value	References
*r*	$15	Arifoğlu et al. (2012)
*c*	$3	Arifoğlu et al. (2012)
*s*	$0	Arifoğlu et al. (2012)
*a*	1.43	Cho (2010)

We think that the key parameters to calibrate are $\mu$ and $\alpha$, both describing the shape of the demand function. So, we will set the remaining parameters, i.e. *k*, $cv_d$, $cv_y$, $\rho$, and *w* to a value and find $\mu$ and $\alpha$, accordingly. Of course, our intention is to analyze the effect of varying these set parameter values on the results. Table 3 summarizes the range of values for these selected parameters.

**Table 3:** Parameter values used in numerical experiments

Parameter	Value
*k*	$\left\{ 10^{-5}, 10^{-6}, 10^{-7} \right\}$
*w*	0.05
$cv_D=cv_Y$	$\left\{ 0.5, 1, 2 \right\}$
$\rho$	$\left\{ -0.9, -0.5, 0, 0.5, 0.9 \right\}$

Note that we use only one value for *w*, as we think that the calibration procedure will adjust the results obtained for the demand function. Additionally, relative values for *k* and *w* are important and hence varying only *k* will suffice. Note that *k* value is exogenously taken, since we do not have any information on the availability increasing strategies.

The available data used for calibration is explained below. CDC reports that 2009–2010 influenza season vaccination coverage among all persons aged higher than six months in the United States is 41.2%, which corresponds to 123.3 million people (Centers for Disease Control and Prevention 2015c). Note that the population size under consideration is 299.272 million people. Besides, according to Department of Health and Human Resources’ report CDC’s fiscal year 2010 budget request for Influenza Program is $158,992,000 (Centers for Disease Control and Prevention 2015d).

**Fig. 2:**
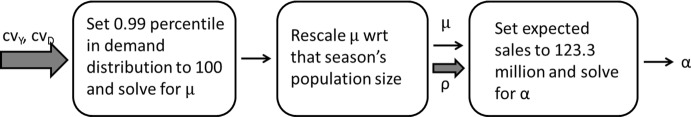
Steps utilized for calibrating parameters of the demand model

The steps of calibration used are briefly illustrated in Fig. 2. Details of procedure are presented in “Appendix 2”. Calibrated $\mu$ and $\alpha$ values for varying values of coefficient of variation of demand, availability, and $\rho$ are tabulated in Tables 8 and 9, respectively in “Appendix 2”.

### Analysis of results

In this section, we use the numerical results obtained as a basis to demonstrate the improvements that can be obtained by applying the proposed joint mechanism. Specifically, we investigate two main issues: (1) improvement in vaccination percentage, and (2) budget savings with joint mechanism, both to get more complete information on the value of applying the joint mechanism. We further analyze two issues: the effect of considering availability uncertainty in this framework and the meaning of the total budget for the policy makers. The details of how computations are made are explained in “Appendix 2”. Note that each numerical problem solved for a given budget level corresponds to a completely different problem environment due to change in demand model’s parameter calibration process, so comparisons among problems may not be consistent. Of course, the problems where the only change is the total budget or *k*, the results are comparable.

#### Improvement in vaccination percentage

One of the reasonable measures to evaluate the performance of joint mechanism is vaccination percentage, which is the ratio of expected sales to population size under consideration. First, we analyze the vaccination percentage to be reached if joint mechanism is applied under that period’s budget amount of 158.992 million. Table 4 reports the outcomes for varying values of *k*, coefficient variation of demand and availability, and $\rho$. The results demonstrate the importance of considering a joint mechanism that eliminates the inefficiencies emanating from both supply and demand sides. The numerical studies show that the vaccination rates of the 2009–2010 season is raised from 41.2% to a range between 42.46% and up to 58.28% depending on the values of *k*, $cv_D$, $cv_Y$, and $\rho$. The gap in the vaccinated percentage between the statistics of 2009–2010 season (41.2%) and the realized percentage under proposed strategy is quite high based on the fact that each percentage corresponds to approximately 2.9 million people. Also, we observe that the improvement in vaccination rates is greater for higher values of efficiency of availability improving strategy (*k*) as expected.

Besides, we compute and tabulate the vaccination rates for a representative set of budget values in Tables 11, 12 and 13 in “Appendix 3”. We observe that unless total budget is very tight, higher uncertainty in the system and negative correlation between demand and availability raise vaccination percentages even more with efficient allocation of budget among intervention alternatives.^1^

**Table 4:** Vaccination percentages achieved by joint mechanism (%), as opposed to reported 41.2%

$cv_D=cv_Y$	*k*	$\rho$		
		− 0.5	0	0.5
0.5	$10^{-5}$	44.99	44.06	42.99
	$10^{-6}$	44.69	43.84	42.86
	$10^{-7}$	43.79	43.18	42.46
1	$10^{-5}$	49.91	47.56	44.99
	$10^{-6}$	48.90	46.81	44.50
	$10^{-7}$	46.11	44.71	43.15
2	$10^{-5}$	58.28	53.46	48.28
	$10^{-6}$	56.18	51.93	47.32
	$10^{-7}$	50.55	47.79	44.76

#### Budget savings with joint mechanism

We further analyze the value of the joint mechanism proposed in terms of total budget spent. Specifically, we determine the budget required to achieve the aforementioned season’s vaccination percentage with the joint mechanism, and demonstrate the savings in the required budget. In Table 5, we present percentage savings for varying values of *k*, $cv_D$, $cv_Y$, and $\rho$. The budget savings ranges between 28.01 and 89.81%. Clearly, applying joint mechanism substantially reduces the required budget to achieve the desired vaccination coverage, especially when uncertainty in the system is low. Also, the budget savings are reasonably high even the availability improving strategy’s efficiency is tenfold lower. Based on the results depicted in Table 5, one can conclude that under higher uncertainty in the system, knowing the correlation would be very significant, of course, if it exists.

**Table 5:** Budget savings relative to current practice (%)

$cv_D=cv_Y$	*k*	$\rho$		
		− 0.5	0	0.5
0.5	$10^{-5}$	87.90	89.10	89.81
	$10^{-6}$	82.45	83.84	84.71
	$10^{-7}$	65.47	67.28	68.55
1	$10^{-5}$	64.89	60.67	51.31
	$10^{-6}$	58.44	54.26	45.12
	$10^{-7}$	40.03	36.16	28.06
2	$10^{-5}$	65.82	61.29	51.37
	$10^{-6}$	59.36	54.86	45.14
	$10^{-7}$	40.90	36.67	28.01

#### What if imperfect availability is approximated by a deterministic function?

Although one can have estimates for the availability’s mean and variance, it may as well be hard to fit a distribution. Thus, while solving these types of decision problems there is a tendency to represent availability by its average value, i.e. in our case this corresponds to a deterministic function of $B_y$. Here, we examine the influence of an imperfect availability without uncertainty on the vaccination percentage.

To obtain the optimal investment amounts ($B_d^d$ and $B_y^d$) for deterministic imperfect availability case, we solve the decision model together with parameter calibration structure by setting availability’s variance to zero. However, availability is uncertain in reality and this affects both the calibration and decision structures (see Tables 8 for $\mu$ values and 10 for updated $\alpha$ values for deterministic imperfect availability case in “Appendix 2”). Using the parameters reflecting availability variability, we evaluate *Q* and expected sales for $B_d^d$ and $B_y^d$ in order to observe the effect of considering deterministic availability. A representative set of results for equal values of $cv_D$ and $cv_Y$, and $k=10^{-6}$ are provided in Table 6, where optimal vaccination percentage found by taking into account availability’s variability and realized percentage with deterministic availability are given in parentheses, respectively. Average loss in vaccination percentages is 0.05%, with the maximum being 0.12%. Comparing the two cases, one can observe that even if the central authority ignores availability uncertainty, it might end up with a vaccination rate in the ballpark of optimal solution by solving calibration and decision problems successively.

**Table 6:** Optimal vs realized vaccination percentages with deterministic availability (%, %)

$\rho , cv$	Budget ($\times 10^6$)							
	30	50	100	150	200	250	300	350
− 0.9, 1	(35.01, 34.96)	(39.18, 39.12)	(45.61, 45.56)	(49.85, 49.80)	(53.10, 53.05)	(55.77, 55.73)	(58.06, 58.01)	(60.06, 60.02)
0, 1	(35.74, 35.73)	(38.79, 38.77)	(43.39, 43.37)	(46.36, 46.35)	(48.61, 48.60)	(50.45, 50.44)	(52.01, 52.00)	(53.37, 53.36)
0.9, 1	(36.19, 36.14)	(37.88, 37.84)	(40.39, 40.36)	(41.99, 41.96)	(43.18, 43.16)	(44.15, 44.12)	(44.96, 44.94)	(45.67, 45.65)
− 0.9, 2	(31.04, 30.94)	(38.06, 37.96)	(49.83, 49.72)	(58.16, 58.04)	(64.82, 64.70)	(70.46, 70.35)	(75.42, 75.30)	(79.85, 79.74)
0, 2	(31.84, 31.82)	(37.05, 37.02)	(45.39, 45.36)	(51.06, 51.03)	(55.49, 55.46)	(59.18, 59.15)	(62.36, 62.34)	(65.19, 65.17)
0.9, 2	(32.39, 32.29)	(35.24, 35.16)	(39.63, 39.56)	(42.52, 42.45)	(44.71, 44.65)	(46.51, 46.46)	(48.05, 47.99)	(49.39, 49.34)

#### Which total budget is sufficient?

The effect of spending more is obviously going to result in more people being vaccinated. Given the structure of the objective function as well as the strategies considered, one may be interested in the effect of any additional budget on the outcome. Table 7 summarizes $ spent per additional person vaccinated using an additional budget of $$\$ 10\times 10^6$$ (i.e. if total budget is $$\$160\times 10^6$$, then we record $ spent per additional person vaccinated with the additional $$\$10\times 10^6$$ budget used over $$\$150\times 10^6$$) for different values of $$cv_D$$, $$cv_Y$$, $$\rho$$, and $$k=10^{-6}$$.

**Table 7:** $ Spent per additional person vaccinated with the additional budget of $$\$10\times 10^6$$

$cv_D,cv_Y$	$\rho$	Total Budget $(\times 10^6)$				
		160	170	180	190	200
0.5	$-$ 0.5	24.49	25.95	27.39	28.83	30.26
	0	34.45	36.54	38.61	40.68	42.73
	0.5	57.47	61.03	64.58	68.12	71.64
1	$-$ 0.5	5.43	5.70	5.98	6.24	6.51
	0	6.75	7.11	7.45	7.80	8.13
	0.5	8.99	9.47	9.95	10.42	10.88
2	$-$ 0.5	2.73	2.85	2.97	3.08	3.19
	0	3.47	3.63	3.79	3.94	4.10
	0.5	4.76	4.99	5.22	5.45	5.67

Note that the results given above represent the additional budget spent to the cost of vaccine paid by the consumer. Comparing these numbers will be a good indication for the policy makers. At 150 million budget, the money spent per person ranges between $0.86 and $1.20 depending on $$cv_D$$, $$cv_Y$$, and $$\rho$$. However, we see that the additional budget spent per person can be considerably high for some cases due to concave structure of demand and availability models. For the cases where the budget spent per additional person vaccinated is higher than $15, the policy maker may prefer to subsidize whole cost of vaccines instead of investing in demand and availability increasing strategies. Moreover, we can interpret whether the budget used in current practice is reasonable based on these results. If uncertainty in the system is high, the current budget used in practice seems to be reasonable and can be increased further; otherwise for low uncertainty case a much lower budget looks to be sufficient for investing in demand and availability increasing strategies.

## Conclusion

We organize the conclusions following the two aspects emphasized in the study: (1) technical background and analysis of the results obtained by the case and (2) issues regarding the applicability of the proposed model.

Inspired from influenza vaccines, this study suggests a model to design intervention mechanism for a public-interest good facing availability issues in order to achieve socially desirable consumption/usage level. In particular, we consider a system composed of a manufacturer and a central planner that intervenes to the system through demand and availability increasing strategies. The goal of the intervention is to resolve the inefficiencies like insufficient demand and availability issues that are inherent to the system and motivate the channel to take socially more acceptable decisions. We develop and incorporate lognormal demand and availability models to our decision problem, general enough to be used in different contexts as well. For the detailed analysis of the model, we follow a pessimistic view and use demand and availability functions with equal coefficient of variation values. Of course, the value of the mechanism will be more if the variances are also affected from the investments made.

The main contribution of this study is to emphasize and technically show that strategies only targeting one aspect of the problem are likely to fall short in an application, emphasizing the importance of coordinating intervention strategies. We verify our results by implementing the proposed approach for the case in US influenza vaccine market. Along with the numerical study, we show that the vaccination coverage in US can be increased considerably via the proposed joint mechanism by addressing both demand and availability issues in the system. Note that in addition to enhancing social welfare, proposed mechanism decreases total budget requirements for a desired level of vaccination. Note that the budget used for improving the vaccination percentages and availability are generally separate as they are funded by different organizations or resources. The budget we used in the numerical study is mainly to support and extend the reach of influenza vaccines. However, this study might provide an incentive to redesign the current fundings under an aggregate budget and to decide on the allocation of budget by a central planner.

We think the model has a considerable potential of applicability, if donor organizations for such programs accept the lead of one central authority (for vaccine case implemented for the world scale, WHO seems to be the natural candidate) for use of the budget. Of course, each organization may propose limits of their donation used for different strategies. (Note that these limits will lead to additional constraints on various budgets; however do not create any difficulties in obtaining the solution. Nevertheless, the obtained optimal solution may not be as good as the unconstrained version similar to the one solved in this work, depending on the severity of the limitations imposed.)

A second possibly debatable issue would be the selection of the objective function. Note that this objective function should more-or-less reflect the targets of the donors, as well. In our case study, we considered the expected number of vaccines sold. Technically, the only requirement for the objective function is to have it a strictly concave function, which is not a difficulty. However, if one selects an increasing function (as we did in our Case study) the optimal value obtained should be reconsidered to make sure that it is acceptable. For instance, if we had reached vaccination levels of 85% or higher in our results, additional considerations may have been inevitable to include in the model to prevent side effects of such high percentages.

Once the major organizational hurdle is removed and an objective function is set, the remaining issues for applicability is more technical. The distributions used in this work are fairly general and can accommodate a large spectrum of data structures. The model requires aggregate data, and hence it is important to be consistent while aggregating the more detailed data and/or estimating data. The procedure we describe in Sect. 5.1 and “Appendix 2” is robust as it makes use of calibrations for those parameters which are not easy to estimate.

Note that some of the technical aspects mentioned in Sect. 3 allows the problem to be transformed to a standard non-linear program which can be solved by a standard package. Also additional properties shown in Sect. 4 and numerical results obtained suggest that certain details modeled are not necessarily important in every case (such as replacing imperfect yield distribution with its expected value and use it as a deterministic parameter). However, unless there is evidence on the contrary, we suggest to start with full detailed model and continue by removing some details when numerical results allow.

Finally, we suggest the central authority to consider relevant what-if type questions to be answered as well as any sensitivity type analyses to be carried out before decisions are made and implemented. This would not be difficult as running non-linear programs is straightforward.

## Appendices

### Appendix 1: Proofs and derivations

**Analysis of lower level problem**

We can rewrite the manufacturer’s profit and expected profit functions as follows:

$$\begin{aligned} P(Q,B_d,B_y)= & {} (r-s) min\left\{ Y(B_y)Q(B_d,B_y),D(B_d)\right\} \\&-cQ(B_d,B_y)+sY(B_y)Q(B_d,B_y)\\ E[P(Q,B_d,B_y)]= & {} (r-s) E[min\left\{ Y(B_y)Q(B_d,B_y),D(B_d)\right\} ]\\&-cQ(B_d,B_y)+sE[Y(B_y)]Q(B_d,B_y) \end{aligned}$$

$E[min\left\{ Y(B_y)Q(B_d,B_y),D(B_d)\right\} ]$ is given by

20 $$\begin{aligned}&\int _{0}^{\infty } \left\{ \int _{0}^{Y(B_y)Q(B_d,B_y)} xf^{B_d}_{D(B_d)|Y(B_y)}(x|y)dx \right. \nonumber \\&\quad \left. + \int _{Y(B_y)Q(B_d,B_y)}^{\infty } Y(B_y)Q(B_d,B_y) f^{B_d}_{D(B_d)|Y(B_y)}(x|y)dx\right\} g^{B_y}(y)dy \end{aligned}$$

Note that $f^{B_d}(.)$ and $g^{B_y}(.)$ are pdfs of demand and availability, respectively. The pdfs are dependent on the investment amounts allocated to improve demand and availability.

First derivative of expected sales is given by

21 $$\begin{aligned}&\frac{\partial E\left[ min\left\{ Y(B_y)Q(B_d,B_y),D(B_d)\right\} \right] }{\partial Q(B_d,B_y)}\nonumber \\&\quad =\int _{0}^{\infty }\int _{Y(B_y)Q(B_d,B_y)}^{\infty }\left\{ f^{B_d}_{D(B_d)|Y(B_y)}(x|y)dx\right\} y g^{B_y}(y)dy \end{aligned}$$

22 $$\begin{aligned}&\quad =E\left[ Y(B_y)1_{\left\{ D(B_d)>Y(B_y)Q(B_d,B_y)\right\} }\right] \end{aligned}$$

Utilizing (22) one can write the first derivative of expected profit and equate it to zero, and derive the first order optimality condition as in (25).

23 $$\begin{aligned}&\frac{\partial E[P(Q,B_d,B_y)]}{\partial Q(B_d,B_y)}=(r-s)E\left[ Y(B_y)1_{\left\{ D(B_d)>Y(B_y)Q(B_d,B_y)\right\} }\right] +sE[Y(B_y)]-c \end{aligned}$$

24 $$\begin{aligned}&=rE[Y(B_y)]-(r-s)E\left[ Y(B_y)1_{\left\{ D(B_d)\le Y(B_y)Q(B_d,B_y)\right\} }\right] -c \end{aligned}$$

25 $$\begin{aligned}&E\left[ Y(B_y)1_{\left\{ D(B_d)\le Y(B_y)Q(B_d,B_y)\right\} }\right] =\frac{rE[Y(B_y)]-c}{r-s} \end{aligned}$$

**Derivation of**$$\pmb{E[Y(B_y)1_{\left\{ D(B_d)\le Y(B_y)Q(B_d,B_y)\right\} }]}$$

Theorem 2.1 of Lien and Balakrishnan (2006) gives an explicit expression for moments of the form $E[X^m Y^n I_{XY}(a,b,K)]$, where $(X,Y)^T$ have bivariate lognormal distribution and $I_{XY}(a,b,K)$ is an indicator function which takes value of 1 when $X^a Y^b \le K$ and 0 otherwise. After applying this theorem to our case with $X=Y$, $Y=D$, $m=1$, $n=0$, $b=1$, $a=-1$, $K=Q$, $\mu _X=ln(H_Y(B_y))-\sigma _Y^{2}$, we obtain the form given in (12).

#### *Proof of Remark 3*

Differentiating (13) with respect to $B_y$, and using the fact that $H_Y (B_y)$ is increasing in $B_y$, we obtain the following:

$$\begin{aligned} \frac{\partial Q(B_d,B_y)}{\partial B_y}&= e^{\left[ \Phi ^{-1}\left( \frac{rH_Y(B_y)-c}{(r-s)H_Y(B_y)}\right) \sqrt{\sigma _{Y}^{2} +\sigma _{D}^2-2\rho \sigma _Y\sigma _D}-\ln (H_{Y}(B_y))+\ln (H_D(B_d))-\sigma _{Y}^2/2 -\sigma _D^{2}/2+\rho \sigma _Y\sigma _D\right] }\\&\quad \times \frac{H_Y (B_y)'}{H_Y (B_y)} \left( \frac{1}{\phi (\Phi ^{-1}\left( \frac{rH_Y(B_y)-c}{(r-s)H_Y(B_y)}\right) } \frac{c}{(r-s)H_{Y}(B_y)} \sqrt{\sigma _{Y}^{2}+\sigma _{D}^2-2\rho \sigma _Y\sigma _D} -1 \right) \end{aligned}$$

For deriving the condition that will make $\frac{\partial Q(B_d,B_y)}{\partial B_y} > 0$, it suffices to find out when $\left( \frac{1}{\phi (\Phi ^{-1}(\frac{rH_Y(B_y)-c}{(r-s)H_Y(B_y)})} \frac{c}{(r-s)H_{Y}(B_y)} \sqrt{\sigma _{Y}^{2}+\sigma _{D}^2-2\rho \sigma _Y\sigma _D} -1 \right) >0$.

Note that $H_Y (B_y)$ takes the value of at most 1 and $\phi (.)$ takes the value of at most 0.3989. Thus, when $\sqrt{\sigma _{Y}^{2}+\sigma _{D}^2-2\rho \sigma _Y\sigma _D}> 0.3989 \left( \frac{r-s}{c} \right)$, the result follows.

**Derivation of**$$\pmb{E[min \left\{ Y(B_y)Q(B_d,B_y),D(B_d) \right\} ]}$$

$$\begin{aligned}&E\left[ min \left\{ Y(B_y)Q(B_d,B_y), D(B_d) \right\} \right] = E\left[ D(B_d) 1_{D(B_d) \le Y(B_y)Q(B_d,B_y)}\right] \\&\quad + E\left[ Y(B_y)Q(B_d,B_y) 1_{D(B_d)\ge Y(B_y)Q(B_d,B_y)}\right] . \end{aligned}$$

As in the derivation of $E[Y(B_y)1_{\left\{ D(B_d)\le Y(B_y)Q(B_d,B_y)\right\} }]$, we apply Theorem 2.1 of Lien and Balakrishnan (2006) in order to derive explicit expressions for the two terms found in the rhs of the equation given above.

$E[D(B_d) 1_{D(B_d) \le Y(B_y)Q(B_d,B_y)}]$ is found by applying the theorem with the following values: $X=D$, $Y=Y$, $m=1$, $n=0$, $a=1$, $b=-1$, $K=Q$, $\mu _X=ln(H_D(B_d))-\sigma _D^{2}$.

$E[Y(B_y)Q(B_d,B_y) 1_{D(B_d)\ge Y(B_y)Q(B_d,B_y)}]$ is derived with the values of $X=D$, $Y=Y$, $m=0$, $n=1$, $a=1$, $b=-1$, $K=Q$, $\mu _X=ln(H_D(B_d))-\sigma _D^{2}$. $\square$

#### *Proof of Proposition 1*

The result follows from the facts that *Q* increases as $B_d$ and $B_y$ increase (by Equation (5)) and utility is an increasing concave function of *Q*. Therefore, central authority will allocate all budget between $B_d$ and $B_y$.

**Derivation of**$$\pmb{E[min \left\{ E[Y(B_y)]Q(B_d,B_y), D(B_d) \right\} ]}$$

26 $$\begin{aligned}&E[min \left\{ E[Y(B_y)]Q(B_d,B_y), D(B_d) \right\} ]=\int _{0}^{E[Y(B_y)]Q(B_d,B_y)} x f(x)dx \nonumber \\&\qquad +\int _{E[Y(B_y)]Q(B_d,B_y)}^{\infty } E[Y(B_y)]Q(B_d,B_y) f(x) dx\nonumber \\&\quad = E[D(B_d)1_{D(B_d) \le E[Y(B_y)]Q(B_d,B_y)}]\nonumber \\&\qquad +E[Y(B_y)]Q(B_d,B_y)(1-F(E[Y(B_y)]Q(B_d,B_y))) \end{aligned}$$

After setting values of applying $X=D$, $Y=Y$, $m=1$, $n=0$, $a=1$, $b=0$, $K=E[Y]Q$, $\mu _X=ln(H_D(B_d))-\sigma _D^{2}$ in Theorem 2.1 of Lien and Balakrishnan (2006), we find the expression given below:

27 $$\begin{aligned}&E\left[ D(B_d)1_{D(B_d) \le E[Y(B_y)]Q(B_d,B_y)}\right] \nonumber \\&\quad =H_D(B_d)\Phi \left( \frac{ln(E[Y(B_y)]Q(B_d,B_y))-ln(H_D(B_d))-\sigma _D^2}{\sigma _D}\right) \end{aligned}$$

Then, substituting $F(E[Y(B_y)]Q(B_d,B_y))=\Phi \left( \frac{ln(E[Y(B_y)]Q(B_d,B_y))-ln(H_D(B_d))+\sigma _D^2}{\sigma _D} \right)$ and (27) into (26), we obtain the expression given in (19). $\square$

#### *Proof of Proposition 2*

Let $T=ln(H_Y(B_y)Q(B_d,B_y))-ln(H_D(B_d))$, then the functions can be expressed as follows:

28 $$\begin{aligned}&E[min \left\{ Y(B_y)Q(B_d,B_y), D(B_d) \right\} ] \nonumber \\&\quad = H_D(B_d)\Phi \left( \frac{T-(\sigma _D-\sigma _Y)^2/2+(\rho -1)\sigma _D \sigma _Y}{\sqrt{(\sigma _D-\sigma _Y)^2+2(1-\rho )\sigma _D \sigma _Y}}\right) \nonumber \\&\qquad +H_Y(B_y)Q \Phi \left( \frac{-T-(\sigma _D-\sigma _Y)^2/2+(\rho -1)\sigma _D \sigma _Y}{\sqrt{(\sigma _D-\sigma _Y)^2+2(1-\rho )\sigma _D \sigma _Y}}\right) \end{aligned}$$

29 $$\begin{aligned}&E[min \left\{ E[Y(B_y)]Q(B_d,B_y), D(B_d) \right\} ] \nonumber \\&\quad = H_D(B_d)\Phi \left( \frac{T-\sigma _D^2/2}{\sigma _D}\right) +H_Y(B_y)Q(B_d,B_y) \Phi \left( \frac{-T-\sigma _D^2/2}{\sigma _D}\right) \end{aligned}$$

Note that the following conditions always hold:

30 $$\begin{aligned} \sqrt{(\sigma _D-\sigma _Y)^2+2(1-\rho )\sigma _D \sigma _Y}>\sigma _D\quad \text{ if }\quad \sigma _Y&>2\rho \sigma _D \end{aligned}$$

31 $$\begin{aligned} (\sigma _D-\sigma _Y)^2/2+(1-\rho )\sigma _D \sigma _Y> \sigma _D^2/2\quad \text{ if }\quad \sigma _Y&>2\rho \sigma _D \end{aligned}$$

Using the expressions (28) and (29), and conditions (30) and (31), we obtain the following relations between the functions:

32 $$\begin{aligned}&E[min \left\{ E\left[ Y(B_y)]Q(B_d,B_y), D(B_d) \right\} \right] \nonumber \\&\quad =E\left[ min \left\{ Y(B_y)Q(B_d,B_y), D(B_d) \right\} \right] \quad \text{ if }\quad \sigma _Y=2\rho \sigma _D \end{aligned}$$

33 $$\begin{aligned}&E\left[ min \left\{ E[Y(B_y)]Q(B_d,B_y), D(B_d) \right\} \right] \nonumber \\&\quad>E\left[ min \left\{ Y(B_y)Q(B_d,B_y), D(B_d) \right\} \right] \quad \text{ if }\quad \sigma _Y>2\rho \sigma _D\end{aligned}$$

34 $$\begin{aligned}&E\left[ min \left\{ E[Y(B_y)]Q(B_d,B_y), D(B_d) \right\} \right] \nonumber \\&\quad<E\left[ min \left\{ Y(B_y)Q(B_d,B_y), D(B_d) \right\} \right] \quad \text{ if }\quad \sigma _Y<2\rho \sigma _D \end{aligned}$$

$$\square$$

## Appendix 2: Details of the computations and calibration for the parameters of the demand model

### Details of calibration for the parameters of the demand model

The calibration method is briefly described in Fig. 2. First two steps of the calibration are reiterated for each different pair of coefficient of variation of demand and availability, whereas last step is repeated for each coefficient of variation value of demand and availability, as well as $\rho$. Firstly, following the value of population size used in Arifoğlu et al. (2012)’s numerical study, i.e. $N=100$, we assume that 0.99 percentile in demand distribution corresponds to 100 vaccines, and obtain mean demand before investment, $H_D(B_d=0)$ or $\mu$. Then, we rescale $\mu$ along with the assumption that total population size of 299.272 million corresponds to 100 people. Lastly, we obtain $\alpha$ by setting expected sales to that aforementioned season’s vaccination level. Note that last step is solved under the observation that all budget is invested in demand increasing strategies.

### Calibrated values for the parameters of the demand model

See Tables 8, 9 and 10.

**Table 8:** $$\mu$$

$(cv_D,cv_Y)$	$\mu (\times 10^3)$
(0.5, 0.5)	111,497.6
(1, 1)	61,014.7
(2, 2)	34,980.2

**Table 9:** $$\alpha$$

$cv_D=cv_Y$	$\rho$				
	− 0.9	− 0.5	0	0.5	0.9
0.5	0.13	0.12	0.11	0.09	0.06
1	0.24	0.23	0.21	0.19	0.17
2	0.36	0.33	0.29	0.25	0.22

**Table 10:** $\alpha$ when yield is assumed to be deterministic

$cv_D$	$\alpha$
0.5	0.08
1	0.19
2	0.25

### Details of the computations

We solve the model given in Sect. 3.2 by replacing (5) with (13) using the nonlinear solver CONOPT within the GAMS environment. We also tested the model with different nonlinear solvers in GAMS environment that can solve logarithmic functions, like KNITRO. However, we observe that CONOPT produces more reliable results. The problems are solved very fast like within approximately 0.003 seconds. We also verify whether the solution obtained is optimal by enumerating the problem presented in Sect. 4.2.

## Appendix 3: Numerical results

See Tables 11, 12, 13.

**Table 11:** Vaccination percentages when $cv_D=cv_Y=0.5$ (%)

$\rho$	*k*	Budget ($\$ \times 10^6$)							
		30	50	100	150	200	250	300	350
− 0.9	$10^{-5}$	41.94	43.02	44.58	45.55	46.27	46.84	47.33	47.74
	$10^{-6}$	41.23	42.44	44.14	45.18	45.94	46.54	47.04	47.47
	$10^{-7}$	39.38	40.82	42.84	44.05	44.93	45.61	46.17	46.66
− 0.5	$10^{-5}$	41.94	42.82	44.09	44.88	45.46	45.92	46.30	46.64
	$10^{-6}$	41.34	42.34	43.73	44.57	45.18	45.67	46.07	46.42
	$10^{-7}$	39.77	40.98	42.65	43.64	44.35	44.91	45.36	45.75
0	$10^{-5}$	41.86	42.50	43.41	43.97	44.38	44.71	44.98	45.21
	$10^{-6}$	41.42	42.15	43.15	43.75	44.19	44.53	44.81	45.06
	$10^{-7}$	40.23	41.13	42.36	43.07	43.58	43.98	44.30	44.58
0.5	$10^{-5}$	41.66	42.06	42.61	42.95	43.19	43.38	43.54	43.68
	$10^{-6}$	41.39	41.84	42.45	42.81	43.07	43.27	43.44	43.59
	$10^-7$	40.64	41.20	41.96	42.40	42.70	42.94	43.13	43.30
0.9	$10^{-5}$	39.38	40.82	42.84	44.05	44.93	45.61	46.17	46.66
	$10^{-6}$	41.16	41.36	41.63	41.79	41.90	41.99	42.06	42.13
	$10^{-7}$	40.87	41.11	41.44	41.63	41.76	41.86	41.94	42.01

**Table 12:** Vaccination percentages when $cv_D=cv_Y=1$ (%)

$\rho$	*k*	Budget ($\$ \times 10^6$)							
		30	50	100	150	200	250	300	350
− 0.9	$10^{-5}$	36.81	40.77	46.96	51.07	54.24	56.85	59.08	61.05
	$10^{-6}$	35.01	39.18	45.61	49.85	53.10	55.77	58.06	60.06
	$10^{-7}$	31.17	35.34	42.03	46.48	49.90	52.71	55.10	57.20
− 0.5	$10^{-5}$	36.89	40.39	45.80	49.37	52.10	54.35	56.26	57.95
	$10^{-6}$	35.34	39.03	44.66	48.35	51.16	53.45	55.42	57.13
	$10^{-7}$	32.01	35.74	41.63	45.52	48.49	50.91	52.97	54.77
0	$10^{-5}$	36.96	39.84	44.25	47.13	49.32	51.11	52.64	53.97
	$10^{-6}$	35.74	38.79	43.39	46.36	48.61	50.45	52.01	53.37
	$10^{-7}$	33.15	36.25	41.09	44.24	46.62	48.55	50.20	51.63
0.5	$10^{-5}$	36.89	39.12	42.49	44.67	46.31	47.65	48.78	49.77
	$10^{-6}$	36.07	38.42	41.92	44.16	45.85	47.22	48.38	49.39
	$10^-7$	34.39	36.77	40.43	42.80	44.58	46.01	47.23	48.28
0.9	$10^{-5}$	36.57	38.21	40.66	42.22	43.40	44.35	45.15	45.85
	$10^{-6}$	36.19	37.88	40.39	41.99	43.18	44.15	44.96	45.67
	$10^{-7}$	35.61	37.25	39.77	41.41	42.63	43.63	44.46	45.19

**Table 13:** Vaccination percentages when $cv_D=cv_Y=2$ (%)

$\rho$	*k*	Budget ($\$ \times 10^6$)							
		30	50	100	150	200	250	300	350
− 0.9	$10^{-5}$	34.02	40.93	52.52	60.72	67.30	72.88	77.78	82.17
	$10^{-6}$	31.04	38.06	49.83	58.16	64.82	70.46	75.42	79.85
	$10^{-7}$	25.14	31.59	43.02	51.32	58.03	63.74	68.75	73.25
− 0.5	$10^{-5}$	33.99	40.10	50.17	57.19	62.76	67.45	71.54	75.19
	$10^{-6}$	31.40	37.66	47.94	55.08	60.74	65.49	69.64	73.33
	$10^{-7}$	26.22	32.09	42.22	49.43	55.18	60.02	64.25	68.03
0	$10^{-5}$	33.88	38.93	47.06	52.61	56.95	60.58	63.72	66.50
	$10^{-6}$	31.84	37.05	45.39	51.06	55.49	59.18	62.36	65.19
	$10^{-7}$	27.75	32.75	41.11	46.90	51.45	55.24	58.52	61.43
0.5	$10^{-5}$	33.62	37.49	43.58	47.66	50.81	53.41	55.65	57.62
	$10^{-6}$	32.24	36.25	42.52	46.69	49.90	52.55	54.82	56.83
	$10^-7$	29.58	33.48	39.82	44.11	47.42	50.16	52.50	54.57
0.9	$10^{-5}$	33.00	35.79	40.10	42.93	45.10	46.88	48.40	49.73
	$10^{-6}$	32.39	35.24	39.63	42.52	44.71	46.51	48.05	49.39
	$10^{-7}$	31.53	34.24	38.59	41.50	43.73	45.56	47.12	48.49

## Footnotes

1 Note that cases with different $$\rho$$’s and coefficient of variation values are not comparable, as the calibration procedure utilized yields different parameter values. We can see the effect of calibration under very low budget values. However, the calibration procedure seems to work well under higher budget levels which are closer to real values used in practice. For instance, in the case where $$cv_D=cv_Y=0.5$$ and budget is 30 million $, the vaccination percentage first increases when $$\rho$$ rises from − 0.9 to − 0.5 and then starts to decrease with more increase in the value of $$\rho$$. On the other hand, vaccination percentage increases with $$\rho$$ at higher budget values.
